# Adult Protective Services’ Intervention on Elder Abuse: Using Standardized Measures for Outcomes

**DOI:** 10.1093/geroni/igaa057.2319

**Published:** 2020-12-16

**Authors:** Pi-Ju Liu, Kendon Conrad, Kathleen Wilber

## Abstract

Adult Protective Services (APS) investigates and substantiates vulnerable adult abuse, neglect, and exploitation (ANE) cases. The frontline social service agency also refers or provides needed services to ANE victims. Outcomes of APS has been scarce, with definitions of outcomes varying from study to study. Using a pretest-posttest design, we partnered with San Francisco and Napa APS to measure changes of ANE harm from case investigation (pretest) to case closure (posttest) using standardized measures called the Identification, Services, and Outcomes (ISO) Matrix. Forty-five APS supervisors and caseworkers used the ISO Matrix on 2,063 cases during the six-month pilot demonstration. Dr. Pi-Ju (Marian) Liu will examine findings on changes of ANE harm and APS services that effectively decreased ANE harm. Responding to 2020’s Annual Scientific Meeting theme “Turning 75: Why Age Matters”, ANE harm and APS services will be compared between younger APS clients age 18-64 and older ones above the age of 65. Dr. Zachary Hass will discuss allegation, abuse severity assessment, services provided, and outcomes across racial and language groups. Dr. Kendon Conrad will present reliability and validity of the ISO Matrix and a shorter version useful for APS practice. Ms Sara Stratton will review unusual cases with outlier ISO Matrix scores to inform researchers’ implementation and practitioners’ use of standardized measures. Dr. Kathleen Wilber, our discussant, will reflect on the use of standardized measures in APS and its impact on both practice and research based on the four presentations.

